# Comparative Performance of Deep Learning and Radiologists for the Diagnosis and Localization of Clinically Significant Prostate Cancer at MRI: A Systematic Review

**DOI:** 10.3390/life12101490

**Published:** 2022-09-26

**Authors:** Christian Roest, Stefan J Fransen, Thomas C Kwee, Derya Yakar

**Affiliations:** Medical Imaging Center, Departments of Radiology, Nuclear Medicine and Molecular Imaging, University Medical Center Groningen, University of Groningen, Hanzeplein 1, 9700 RB Groningen, The Netherlands

**Keywords:** prostatic neoplasms, magnetic resonance imaging, deep learning

## Abstract

Background: Deep learning (DL)-based models have demonstrated an ability to automatically diagnose clinically significant prostate cancer (PCa) on MRI scans and are regularly reported to approach expert performance. The aim of this work was to systematically review the literature comparing deep learning (DL) systems to radiologists in order to evaluate the comparative performance of current state-of-the-art deep learning models and radiologists. Methods: This systematic review was conducted in accordance with the 2020 Preferred Reporting Items for Systematic Reviews and Meta-Analyses (PRISMA) checklist. Studies investigating DL models for diagnosing clinically significant (cs) PCa on MRI were included. The quality and risk of bias of each study were assessed using the checklist for AI in medical imaging (CLAIM) and QUADAS-2, respectively. Patient level and lesion-based diagnostic performance were separately evaluated by comparing the sensitivity achieved by DL and radiologists at an identical specificity and the false positives per patient, respectively. Results: The final selection consisted of eight studies with a combined 7337 patients. The median study quality with CLAIM was 74.1% (IQR: 70.6–77.6). DL achieved an identical patient-level performance to the radiologists for PI-RADS ≥ 3 (both 97.7%, SD = 2.1%). DL had a lower sensitivity for PI-RADS ≥ 4 (84.2% vs. 88.8%, *p* = 0.43). The sensitivity of DL for lesion localization was also between 2% and 12.5% lower than that of the radiologists. Conclusions: DL models for the diagnosis of csPCa on MRI appear to approach the performance of experts but currently have a lower sensitivity compared to experienced radiologists. There is a need for studies with larger datasets and for validation on external data.

## 1. Introduction

Prostate cancer (PCa) continues to be a major public health problem, with an estimated 1,400,000 new cases and 375,000 deaths worldwide in 2020 [1]. Clinically significant (cs, defined as Gleason Grade Group > 1) PCa is associated with significantly worse survival and an increased risk for developing metastases [2]. Therefore, accurate methods for the detection and characterization of PCa are critical for the selection of an appropriate treatment plan. Magnetic resonance imaging (MRI) is a commonly used noninvasive modality for the detection of csPCa. The PI-RADS (v2) guidelines were introduced as a standardized system for reading and reporting findings at prostate MRI and are widely used by radiologists in clinical practice [3]. Studies have shown a high sensitivity and moderate specificity for PI-RADS [4]. However, the ability of PI-RADS to detect csPCa is highly dependent on reader experience and has limited reproducibility due to high intra- and inter-reader variability [5,6,7].

Artificial intelligence (AI) has the potential to revolutionize medical image analysis by automating manual tasks, increasing diagnostic performance, and reducing the costs of healthcare. Until recently, research on diagnostic AI applications in medical imaging primarily focused on radiomics in combination with traditional AI algorithms [8]. However, studies showed that these systems have several limitations, including limited diagnostic capabilities, poor generalization to external datasets, and a dependency on expert input [9,10]. In recent years, focus has shifted away from radiomics towards deep learning (DL), fueled by an increasing availability of high-quality datasets, computing power, and more effective algorithms [8]. Numerous studies have demonstrated that DL can be used to automatically extract diagnostically relevant information from highly complex imaging data [11,12,13].

DL may improve the diagnosis of csPCa on MRI and reduce the need for invasive biopsies. Fully autonomous DL systems for the diagnosis of csPCa on MRI have been reported to approach the diagnostic performance of expert radiologists. The tasks of diagnosis at the patient level and lesion localization in particular have received attention from researchers due to their relevance in clinical practice. Previous reviews and meta-analyses have investigated the diagnostic accuracy of DL models but either did not compare the performance to radiologists or included mainly traditional machine learning techniques [14,15,16,17].

We hypothesized that modern DL systems can achieve a diagnostic performance that is comparable to that of expert radiologists. Therefore, the objective of this systematic review was to provide a comprehensive overview of the available literature comparing the diagnostic performances of DL-based systems and assessments by radiologists for the patient-level diagnosis and localization of csPCa on MRI.

## 2. Materials and Methods

An exhaustive systematic search and review was performed to identify recent literature relevant to diagnostic DL for prostate MRI. For the scope of this study, deep learning was defined as predictive modeling using an artificial neural network consisting of multiple trainable layers. This work was carried out in accordance with the Preferred Reporting Items for Systematic Reviews and Meta-Analysis (PRISMA) guidelines [18]. We have attached the PRISMA checklist in supplementary (Table S1).

### 2.1. Eligibility Criteria

Records were assessed and filtered based on the following criteria: (i) year of publication is after 2013, (ii) focus on the detection or classification of prostate cancer on MRI scans using DL modeling, (iii) a comparison is made between the diagnostic performance of radiologists and the proposed DL system, (iv) size of the combined study cohort is at least 300 patients, (v) full text is available. Articles that did not report the performance of radiologists and AI at the same sensitivity cutoff and did not provide receiver-operating characteristic (ROC) or free-response ROC (FROC) curves from which this could be extracted were excluded. Reports written in languages other than English were excluded.

### 2.2. Search Strategy and Sources

A list of keywords was compiled and used to search Medline, Embase, and Scopus. The following keywords were used to search for studies relevant to the topic: ((Deep learning) OR (Deep Neural Network) OR (DNN) OR (MACHINE LEARNING) OR (AI) OR (ARTIFICIAL INTELLIGENCE)) AND ((prostate) OR (PCA)) AND ((MRI) OR (bpMRI) OR (magnetic resonance imag*)). The relevance of the retrieved records was assessed by a single reviewer (CR., with seven years of experience in DL and 1.5 years of experience in DL in medical imaging) using the inclusion criteria described in Section 2.3. The records were retrieved in December 2021.

### 2.3. Study Quality

The Checklist for Artificial Intelligence in Medical Imaging (CLAIM) [19] was used to assess the scientific quality of the included studies. The CLAIM checklist comprises 41 elements that describe the best practices for AI research in medical imaging and was created with the aim of helping authors and reviewers to evaluate AI manuscripts. It contains separate sections for the title, introduction, methods, results, and discussion. It further divides the methods section into ‘data’, ‘ground truth’, ‘data partitions’, ‘model’, ‘training’, and ‘evaluation’ and divides the results section into ‘data’ and ‘model performance’. Two reviewers independently (C.R. & S.J.F., with one year of experience in DL in medical imaging, under the supervision of an expert uroradiologist [D.Y.] with nine years of experience in prostate MRI) reviewed each of the included studies using CLAIM to determine (1) whether each item was applicable to the study and (2) whether each applicable item was adhered to. Any discrepancies between the ratings were resolved in a deliberation between the reviewers. Since CLAIM does not include a standardized method for determining a final quality score, we quantified the study quality as the percentage of fulfilled checklist items out of all applicable checklist items. A Kruskall–Wallis test and post-hoc Dunn’s test with Bonferroni correction were performed to determine whether the study quality differed between the sections of the checklist. A copy of the CLAIM checklist has been included in the supplementary material (Appendix A). The risk of bias was evaluated for each of the included studies using the QUADAS-2 tool [20].

### 2.4. Study Selection and Data Extraction

Reference files were extracted from the searched databases and added to Mendeley (Version 1.19.8, Elsevier, London, UK). A single reviewer (C.R) checked each title, abstract, and key terms manually for their fit to the specified inclusion criteria. The selected studies were fully read, and a predetermined list of characteristics relating to the study design, patient cohort, and deep learning methodology were extracted (Table 1).

Performance metrics (sensitivity, specificity, and false positives per patient) for the radiologist were extracted for PI-RADS ≥ 3 and PI-RADS ≥ 4 thresholds. For the DL systems, sensitivity was assessed at the same level of specificity or false positives per patient as the respective radiologist benchmarks to enable a direct comparison between the specificity or false positives per patient at an identical sensitivity. When the latter metrics were not explicitly reported, they were estimated from ROC or FROC curves. In the case that an ROC curve was provided for the radiologists instead of explicit PI-RADS thresholds, performance was estimated from the ROC curves at specificity cutoffs derived from the literature (18.5% for PI-RADS ≥ 3 and 67.5% for PI-RADS ≥ 4) [4]. If the results of multiple radiologists were separately reported, performance metrics were extracted for the most senior radiologist. Differences in performance metrics between the radiologists and DL were evaluated for statistical significance using a two-sided paired Wilcoxon’s test per study. *p*-values below 0.05 were considered significant. Averages for the age and serum prostate-specific antigen (PSA) levels of the combined cohort were derived using weighted pooling. Other results were reported using descriptive statistics. Statistical analyses were performed in R version v4.1.0 (R Foundation for Statistical Computing, Vienna, Austria, with the additional packages FSA v0.9.2 (Ogle, 2022) and Hmisc 4.6-0, (Harrell Jr, 2021).

## 3. Results

**Figure 1:**
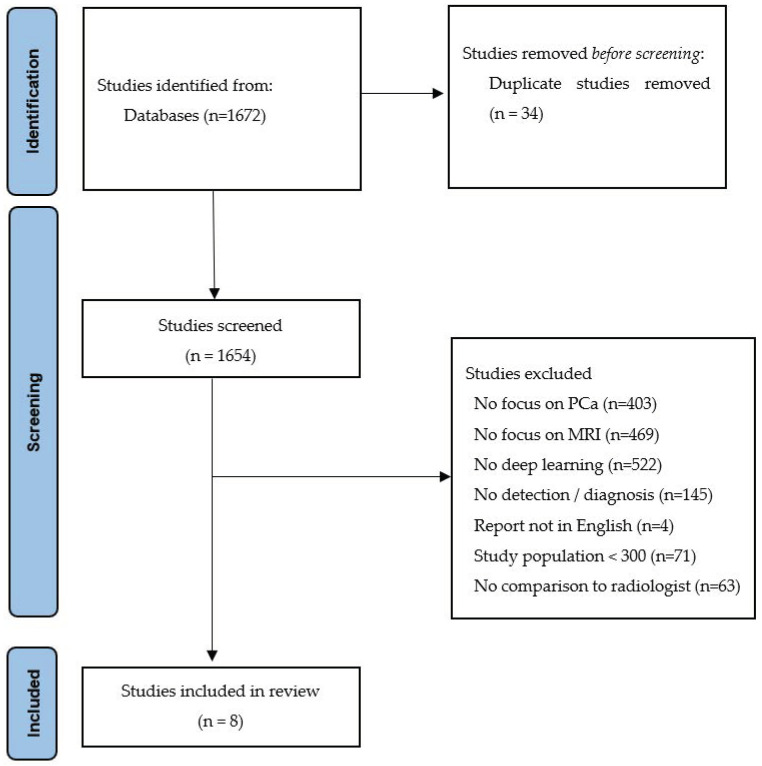
PRISMA 2020 Flow diagram.

An initial search yielded 1672 citations. After the removal of duplicates, 1638 were eligible for the screening of the title and the abstract. The PRISMA flow diagram (Figure 1) shows an overview of the selection process. The results of the bias assessment using QUADAS-2 are shown in Appendix B.

### 3.1. Study Characteristics

Eight studies were included in the final selection [21,22,23,24,25,26,27,28]. An overview of the selected studies is shown in Table 1. An additional manual search to identify potentially missed articles in the references of the included studies did not identify any missed articles. All studies were published in or after 2019. The median pooled age across cohorts was 64 years (IQR 64–65.8). The median pooled PSA was 7.4 mg/L (IQR 7.1–7.85). The median number of included patients was 526 (IQR 413–902). One study ([25]) used a five-point Likert scoring system instead of PI-RADS scores, as diffusion-weighted imaging was not available. An overview of the study characteristics for each study is presented in Table 1. The median level of experience of the most senior radiologist in each study was 15 years (IQR 10.8–17.5). Characteristics of the evaluation strategies and cohorts for each study are presented in Table 2.

#### 3.1.1. MRI

Seven studies (87.5%) evaluated a DL system using bi-parametric MRI (T2-weighted imaging [T2W] + diffusion-weighted imaging [DWI]), and one study (12.5%) used only the T2W sequence. None of the included studies used dynamic contrast-enhanced imaging. Six studies (75%) exclusively used images acquired using 3.0 Tesla scanners, while the remaining two studies (25%) included a mixture of 1.5T and 3.0T scanners. MRI scanner vendors included Siemens Healthineers (seven studies, 87.5%), Philips Healthcare (two studies, 25%), and General Electric Healthcare (one study, 12.5%). Four studies (50%) were performed using multi-center data.

#### 3.1.2. Pathology Annotations

The histopathological grades were obtained exclusively using MRI-targeted biopsies in two studies (25%). Two studies (25%) used grades obtained through radical prostatectomy. The four remaining studies (50%) used a combination of systematic biopsies, MRI-targeted biopsies, and radical prostatectomy (Table 2).

### 3.2. Deep Learning Modeling

The majority of the included studies (n = 5, 62.5%) used a model architecture based on U-Net [29]. The remaining three studies used a convolutional neural network (CNN) classifier. Six studies described DL pipelines that could generate predictions completely autonomously from the MRI input (i.e., without requiring any manual actions to generate predictions). Deniffel et al. [28] required only the rough location of the prostate as a bounding box. Another study by Hiremath [22] processed the scans at lesion level and required lesion segmentations to crop the region-of-interest as the input for the model.

Four studies (50%) used only two-dimensional (2D) operations within their model architecture, while two studies (25%) used three-dimensional (3D) operations. One study [23] used a combination of 2D and 3D architectures. One study used a 2.5 approach [25] by supplying two slices adjacent to the target slice as additional inputs to the model while using 2D operations within the model architecture. Khosravi et al. [21] applied transfer learning to initialize the model’s parameters, using a model that was pretrained on ImageNet [30]. All other studies trained their models from scratch. Cao et al. [25] and Netzer et al. [23] both evaluated the performance of a previously developed algorithm (‘FocalNet’ and ‘nnUNet’, respectively). All but one of the proposed methods provided methods for the visual inspection of the DL predictions, which included prediction heatmaps (five studies, 62.5%), gradient-weighted class activation mapping (Grad-CAM) [22,31], and class-activation maps [21].

### 3.3. Quality of Included Studies

**Figure 2:**
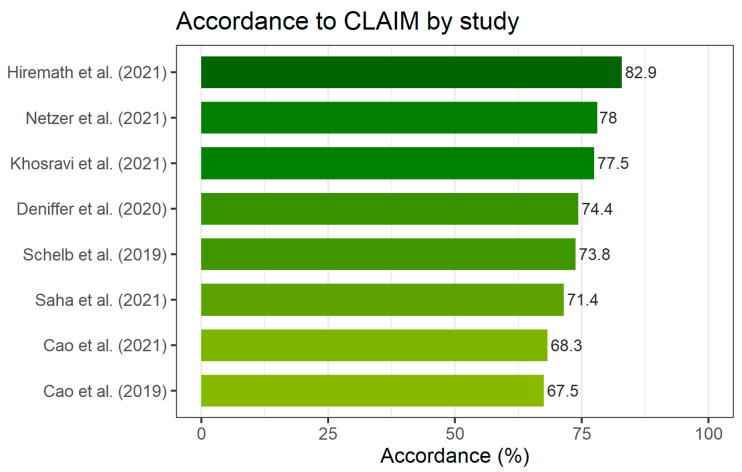
An overview of the quality of the included studies evaluated using the CLAIM checklist. The scores reflect the percentage of applicable checklist items that were sufficiently reported by each of the studies. All scores were agreed upon by two reviewers [21,22,23,24,25,26,27,28].

**Figure 3:**
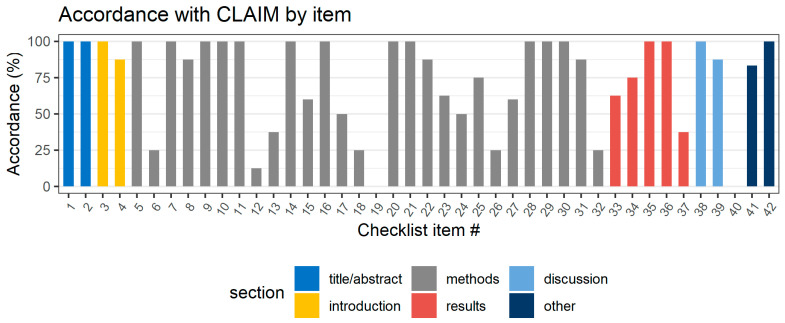
Average accordance with the CLAIM checklist by checklist item. Checklist items that were considered not applicable to the study were omitted from the calculation.

The CLAIM evaluation for each of the studies included in this review is presented in Figure 2. The median accordance with applicable CLAIM items by study was 74.1% (IQR: 70.6–77.6, range: 67.5–82.9). The application of the CLAIM checklist revealed several common weaknesses in the selected studies. Accordance with CLAIM by checklist item is presented in Figure 3. The lowest accordance with CLAIM was found for the intended sample size (item 19), which was not sufficiently reported by any of the included studies. Descriptions of deidentification methods (item 12) were insufficient in seven studies (87.5%). Methods for the mitigation and measurement of intra- and interrater variability (item 18), the selection of the final model (item 26), and the validation on external data (item 32) were insufficiently reported by six studies (75%). The median accordance was the lowest for the reporting of the methods section (69.2%, IQR: 67.9–75) and the highest for the title/abstract (100%, IQR: 100–100). The quality of reporting in the methods sections was significantly lower than that in the introduction (*p* = 0.018) and discussion (*p* = 0.018) sections. The assessment with QUADAS-2 found four studies at risk of bias [23,25,26,28] and two studies with concerns regarding applicability [22,25]. Specifically, a high risk of bias in the index test and reference standard was found in Cao et al. [26] due to the exclusion of MRI invisible lesions from the analysis. Applicability concerns were found for Hiremath et al. [22] because of the indirect comparison between the radiologist and DL as part of a clinical nomogram.

### 3.4. Diagnostic Accuracy at the Patient Level

**Figure 4:**
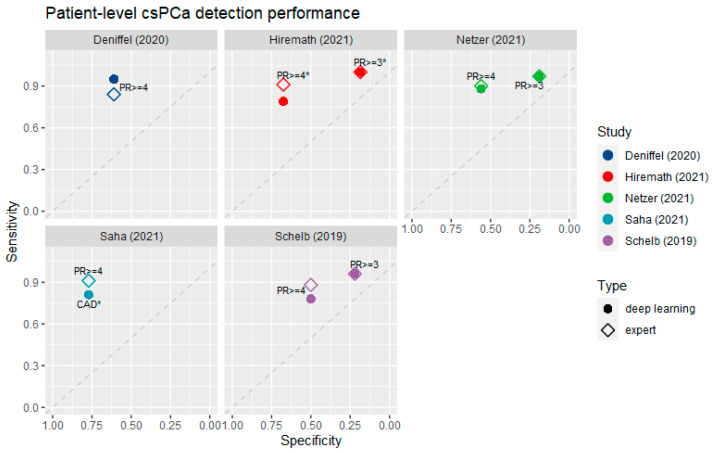
Sensitivity and specificity of DL systems for the diagnosis of csPCa at the patient level, compared to the respective radiologist benchmarks [22,23,24,27,28]. (*) The radiologist’s performance in Hiremath et al. [22] was estimated from ROC curves at specificity estimates derived from the literature [4].

The results of the patient-level analysis are presented in Figure 4. The average AUC for diagnosing csPCa at the patient level by the DL systems was 0.82 (SD = 0.047). At cutoffs of PI-RADS ≥ 3 and PI-RADS ≥ 4, the mean specificity of the radiologists was 19.8% (SD = 1.8%) and 62.3% (SD = 10.4%), respectively. For PI-RADS ≥ 3, the sensitivities of the radiologists and DL were identical at 97.7% (SD = 2.1%). For PI-RADS ≥ 4, the radiologists had an average sensitivity of 88.8% (SD = 3.0%), while the sensitivity of the DL systems was lower at 84.2% (SD = 7.2%). This difference was not significant (*p* = 0.43). In addition, no significant differences in sensitivity or AUC between the radiologists and DL were reported by the included studies.

### 3.5. Lesion Localization Performance

Sensitivities for the DL and radiologists for the localization of csPCa lesions are presented in Table 3. Three studies evaluated the lesion localization performance of the DL models using FROC curves and showed a mean sensitivity at 0.1 false positives per patient of 0.45 (SD = 0.17) and a mean sensitivity at one false positive per patient of 0.82 (SD = 0.14). Radiologists performed better than the respective DL systems at all thresholds. The differences in sensitivity between the DL and radiologists were not evaluated for statistical significance due to the heterogeneity in the reported levels of false positives per patient.

DL detected several lesions that were not detected by radiologists. Cao et al. [25] reported that 5.6% of csPCa lesions detected by their DL model were missed by the best-performing radiologist at 0.44 false positives per patient. Conversely, up to 23.7% of lesions detected by the best radiologist (radiologist #2) were not detected by the DL model. Compared to the least accurate radiologist (radiologist #3) in Cao et al. [25], 13.8% of the lesions detected by DL were missed by the radiologists, while only 10.6% of lesions detected by the radiologist were missed by the AI. Saha et al. [27] reported that their model correctly identified clinically significant lesions in three patients that were missed by four radiologists.

## 4. Discussion

This systematic review reports on studies that compared the diagnostic performance of DL and radiologists for the detection and localization of csPCa on MRI. The final selection included eight studies, which each compared a DL model to clinical assessment using PI-RADS or Likert scores. Our primary finding is that DL achieved a diagnostic performance that was comparable to radiologists, although slightly lower for both patient-level diagnosis and lesion localization tasks. More large-scale studies are needed to further develop DL as a potential alternative to assessment by radiologists in the future. The included studies were evaluated on the quality of the included studies with the CLAIM checklist. We found that the overall quality for the included studies was good (74.1%).

The CLAIM checklist revealed several common weaknesses in the quality of the included studies. In line with the findings of previous systematic reviews on this topic [14,16], we found a lack of validation on external data. Without validation on external data, only limited conclusions can be drawn about the expected performance on data obtained from different scanners and vendors. Secondly, we found that the majority of studies failed to report relevant details pertaining to the DL modeling, particularly regarding the strategy for selecting the final model and the initialization of the model parameters. Incomplete descriptions of methodology negatively impact the reproducibility of the study, and the use of guidelines and checklists (such as the CLAIM checklist) can help authors identify weaknesses in their study design and in the reporting of the results.

Both reviewers agreed that CLAIM was effective at evaluating the completeness of the reporting and recommended its use by authors as a guideline when writing reports; however, we found that, in several cases, evaluation with CLAIM did not accurately reflect the perceived study quality due to overly specific criteria which are rarely completely adhered to.

DL systems demonstrated good performance for the diagnosis of csPCa, achieving an identical patient-level performance to radiologists at a threshold of PI-RADS ≥ 3. The lower sensitivity found for diagnosis at a threshold of PI-RADS > 4 and lesion localization indicated that DL systems are currently performing slightly worse than radiologists. This result can be primarily attributed to the use of small datasets. The performance of DL is highly dependent on the number of images used for training and scales with the size of the dataset. Previous work by Hosseinzadeh et al. [32] demonstrated that global expert-level performance may only become achievable beyond 2000 training samples. In this study, we only included studies with more than 300 patients; yet, the median population size was only 526. We recommend that researchers aim to perform future studies with larger annotated datasets to enable a fair comparison between radiologists and AI. Multiple authors [23,24,28] indicated that the availability of larger annotated datasets would likely lead to improved diagnostic performance. Another factor to consider is that the radiologist benchmarks in the included studies were relatively strong, with the experience of the radiologists ranging from 10 to 18 years, and DL may already outperform less experienced radiologists at local institutions [32].

A shared decision-making strategy between DL and experts may have the potential to improve the detection of csPCa in clinical practice. Three studies evaluated a hybrid classifier that combined DL predictions with radiologist scores. Hiremath et al. [22] developed and validated an integrated nomogram, which combines clinical parameters, PI-RADS, and DL predictions to diagnose csPCa. They showed that the combination of PI-RADS and DL achieved a significantly better diagnostic accuracy than DL and PI-RADS individually. Similarly, Schelb et al. [24] reported a significant improvement in the positive predictive value for PI-RADS ≥ 3 and PI-RADS ≥ 4 detections by using DL to reduce false positive detections. It is important to note that the combination of DL and radiologist predictions was performed automatically in these studies, which takes the final decision out of the hands of the expert. Therefore, these experiments are not completely analogous to concurrent-reader computer-aided diagnosis, which involves a final decision by an expert [8,33]. Studies using such concurrent-reader DL systems are currently needed to show that radiologists’ decisions can be positively influenced by AI systems and to demonstrate a higher level of efficacy for the clinical application of DL in the diagnostic imaging process [34].

The generalizability of AI systems continues to be an important challenge for the application of AI in clinical practice [9]. The ability of models to retain their diagnostic performance when applied to external data is often impaired by their sensitivity to diagnostically irrelevant differences between imaging data obtained from different centers and vendors. When such differences are misinterpreted by the model, they can severely impact diagnostic performance. Previous studies showed that radiomics-based machine learning models are particularly affected by this [9]. Three studies that evaluated their model on external data reported stable performance across internal and external datasets, indicating that DL was relatively robust regarding differences in image acquisition [22,23,27]. This can be partially explained by the extensive regularization strategies that are commonly included in modern DL pipelines to prevent overfitting, such as L2-penalties, weight decay, and data augmentation, which help the model to learn robust features during training [35].

An important role for MRI in PCa is to reduce the number of unnecessary biopsies in patients suspected of harboring csPCa [36,37]. Since DL predictions are given on a continuous scale, they allow for more precise configurability compared to PI-RADS, which is reported on a five-point Likert scale. The ability to threshold predictions at any sensitivity and specificity tradeoff within the diagnostic capabilities of the model may be used to create better biopsy strategies than those based on PI-RADS. Two studies used decision curve analysis to determine whether the number of biopsies could be safely reduced using a biopsy strategy based on DL predictions. In Deniffel et al. [28], decision curve analysis showed that a biopsy strategy based on their calibrated DL model could avoid 3.7 times more unnecessary biopsies compared to strategies based on PI-RADS. Similarly, Hiremath et al. [22] concluded that a combined strategy based on DL and PI-RADS prevented more biopsies compared to strategies based on PI-RADS + clinical parameters alone.

Two studies noted a limited ability of DL to detect smaller csPCa lesions, which requires further investigation [25,27]. According to Cao et al. [25], the sensitivity of the AI was more affected by the size of the lesion than by the aggressiveness of the lesion, indicating that DL models may be biased towards detecting larger lesions. A potential solution could be to adjust the predicted likelihood for smaller lesions during post-processing. The spatial congruity between the DL-generated predictions and radiologist annotations was also lower for smaller lesions, which can be partly explained by their relative sensitivity to minor sequence misalignments and inconsistencies in labelling. This naturally leads to more variance in congruity metrics such as the Dice score [27]. Saha et al. [27] suggests that the use of probabilistic labels during training could potentially help mitigate this problem by conditioning the model to account for labelling variability in its predictions. This variability in spatial congruity for smaller lesions may also affect the comparison for the lesion localization task using FROC analysis, in which a criterion based on a minimum overlap or proximity is used to determine whether a detection by the DL system counts as a true positive or false positive [25,26,27].

In this review, we compared the performance of AI systems to that of radiologists directly using the FP rate and specificity at specific sensitivity cutoffs. In practice, it cannot be guaranteed that a specific trade-off between sensitivity and specificity/FP rates will be maintained over time. To prevent significant divergence from the intended operating point of an AI system, the performance may be tracked and recalibrated periodically after deployment.

This study has limitations. First, the performances of the algorithms were only compared using selected separate performance metrics. Systematic literature reviews by [14,15,16] previously concluded that there is a lack of consistency and clarity in the reporting of performance metrics in diagnostic AI, which hampers attempts to compare the performance of different works. For this reason, we limited our analysis to sensitivity at equivalent levels of specificity and false positives per patient, as these are common practices for reporting diagnostic performance for classification and localization tasks, respectively. Secondly, the assessment of study quality using the CLAIM checklist and guidelines left some room for interpretation; therefore, others may arrive at different CLAIM scores than ours. Third, we only considered studies with at least 300 patients for inclusion in this review, because DL requires large numbers of training samples, and small numbers of training samples are less likely to yield reproducible results [32]. However, this means that the performances reported in our study may not be reflective of those achieved by smaller-scale studies. Fourth, the assessment using QUADAS-2 found multiple included studies at risk for bias, which may limit the generalizability of our results. Fifth, the identification of relevant articles was carried out by a single reviewer. Human error may potentially have resulted in relevant articles being missed, although an additional manual reference search was performed to mitigate this risk. Lastly, the majority of studies included in this review were performed with scans acquired using systems of Siemens Healthineers, which makes our present results potentially less applicable to scanners from different vendors.

In conclusion, DL models for the detection of csPCa on MRI appear to approach the performance of expert radiologists but have a lower sensitivity compared to experienced radiologists for the diagnosis of csPCa at the patient level at a threshold of PI-RADS ≥ 4 and for the localization of csPCa lesions. DL studies have been performed with relatively small datasets so far, and there is a need for studies that evaluate the performance of DL using larger training datasets. Shared decision making between DL and radiologists has shown potential to improve diagnostic performance.

## Figures and Tables

**Table 1:** An overview of the study characteristics for each of the included studies. T2W: T2-weighted imaging, ADC: Apparent Diffusion Coefficient map, DWI: Diffusion-weighted imaging, FPP: False positives per patient [21,22,23,24,25,26,27,28].

Author	Country	No. Pat.	Sequences	Scanner	Vendor	Rad. Exp.	PSA (mg/L)	Age	Scoring Level	DL Approach	Required Expert Input	Interpretability	AUC (Patient Level)	Sens. at 0.1 FPP	Sens. at 1 FPP
Saha et al. [27]	The Netherlands	2732	T2W, ADC, DWI	3T (Trio, Skyra, Prisma)	Siemens	25 yrs	7.85	65.8	Patients, lesion	U-Net	-	Heatmaps	0.862	0.64	0.9
Cao et al. [25]	US	553	T2W, ADC	3T (Trio, Verio, Skyra, Prisma)	Siemens	19 yrs	6.2	62.5	Patient, lesion	U-Net (FocalNet)	-	Heatmaps	-	0.3	0.65
Netzer et al. [23]	Germany	1832	T2W, ADC, DWI	3T (Prisma), 1.5T (Aera)	Siemens	11 yrs	7.1	64.0	Patient, sextant	U-Net (nnUNet)	-	Heatmaps	0.85	-	-
Cao et al. [26]	US	417	T2W, ADC	3T (Trio, Verio, Skyra, Prisma)	Siemens	10 yrs	-	-	Lesion, voxel	U-Net (FocalNet)	-	Heatmaps (by Gleason)	-	0.42	0.9
Deniffel et al. [28]	Canada	499	T2W, ADC, DWI	3T (Achieva)	Philips	15 yrs	7.2	64.4	Lesion	Classifier	Prostate location	-	0.85	-	-
Schelb et al. [24]	Germany	312	T2W, ADC, DWI	3T (Prisma)	Siemens	10 yrs	6.9	64.0	Patient, sextant	U-Net	-	Heatmaps	-	-	-
Hiremath et al. [22]	US	592	T2W, ADC	3T (Trio, Verio, Skyra, Achieva)	Siemens, Philips	15 yrs	6.4	63.9	Lesion	Classifier (AlexNet, DenseNet)	Lesion segmentation	GradCAM	0.76	-	-
Khosravi et al. [21]	US	400	T2W	3T, 1.5T	Siemens, GE	17 yrs	-	-	Patient	Classifier (Inception V1)	-	Class activation maps	0.78	-	-

**Table 2:** An overview of the characteristics of the evaluation strategy and cohort for each of the included studies. TB: MRI-targeted biopsy; SB: systematic biopsy; RP: radical prostatectomy; CV: cross validation. * In combined cohort. ** Included Gleason 3 + 4 = 7 in the definition of a low-risk group.

Author	Description of Test Cohort	Biopsy Method	Train Size	Test Size	csPCa in Test Cohort	Cohort Split	Evaluation Strategy
Saha et al. [27]	Biopsy naive men with elevated PSA	SB; TB (in-bore)	1584 studies	296 patients	86 patients (29%)	institution	held-out test set
Cao et al. [25]	Patients who underwent RP	RP	427 patients	126 patients	114 patients (90%)	temporal	held-out test set
Netzer et al. [23]	Consecutive patients with clinical indication for biopsy	TB	806 studies	682 studies	235 exams (34%)	temporal	held-out test set
Cao et al. [26]	Preoperative patients who later underwent RP	RP	333–334 studies	84–83 studies	442 lesions (61%) *	CV	CV (five-fold)
Deniffel et al. [28]	Patients without prior known csPCa	TB	449 patients	50 patients	19 patients (38%)	random selection	held-out test set
Schelb et al. [24]	Consecutive patients with clinical indication for biopsy	TB; SB	250 patients	62 patients	26 patients (42%)	random selection	held-out test set
Hiremath et al. [22]	Patients with prostate cancer (various datasets)	TB; SB; RP	368 patients	224 patients	199 lesions (63%)	institution	held-out test set
Khosravi et al. [21]	Patients suspected for prostate cancer (various datasets)	TB; SB; RP	243 patients	40 patients	20 patients (50%) **	random selection	held-out test set

**Table 3:** Relative performance of deep learning and radiologists for the localization of csPCa lesions (ISUP > 1) at the same levels of false positives (FP) per patient. (*) Cao et al. [25] reported performances for multiple radiologists; the presented scores are for the best-performing radiologist.

Study	FP Per Patient	Sensitivity (Expert)	Sensitivity (DL)	Difference
Cao et al. [26]	0.625	81%	79%	−2%
Cao et al. [25] *	0.15	48.7%	40.6%	−8.1%
	0.5	65%	52.5%	−12.5%
	1.24	77.5%	65.6%	−11.9%
Saha et al. [27]	0.29	91%	78.5%	−12.5%

**Figure A1:**
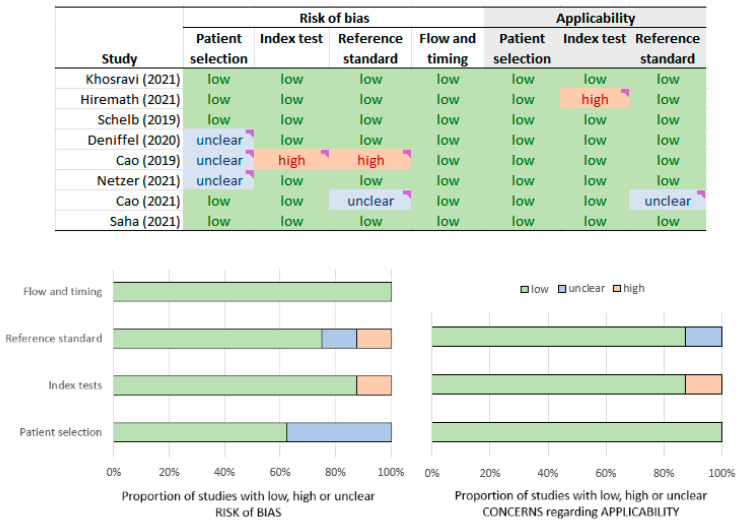
Results of the bias assessment using the QUADAS-2 tool [21,22,23,24,25,26,27,28].

## Data Availability

The authors agree to share the collected data and statistical code upon reasonable request.

## Supplementary Materials

The following supporting information can be downloaded at: https://www.mdpi.com/article/10.3390/life12101490/s1, Table S1: Prisma Checklist.

## Appendix A

**Table A1:** CLAIM: Checklist for Artificial Intelligence in Medical Imaging [19].

Section/Topic	No.	Item
Title/Abstract		
	1	Identification as a study of AI methodology, specifying the category of technology used (e.g., deep learning)
	2	Structured summary of study design, methods, results, and conclusions
Introduction		
	3	Scientific and clinical background, including the intended use and clinical role of the AI approach
	4	Study objectives and hypotheses
Methods		
Study Design	5	Prospective or retrospective study
	6	Study goal, such as model creation, exploratory study, feasibility study, and non-inferiority trial
Data	7	Data sources
	8	Eligibility criteria: how, where, and when potentially eligible participants or studies were identified (e.g., symptoms, results from previous tests, inclusion in registry, patient-care setting, location, dates)
	9	Data pre-processing steps
	10	Selection of data subsets, if applicable
	11	Definitions of data elements, with references to Common Data Elements
	12	De-identification methods
	13	How missing data were handled
Ground Truth	14	Definition of ground truth reference standard, in sufficient detail to allow for replication
	15	Rationale for choosing the reference standard (if alternatives exist)
	16	Source of ground-truth annotations; qualifications and preparation of annotators
	17	Annotation tools
	18	Measurement of inter- and intrarater variability; methods to mitigate variability and/or resolve discrepancies
Data Partitions	19	Intended sample size and how it was determined
	20	How data were assigned to partitions; specify proportions
	21	Level at which partitions are disjoint (e.g., image, study, patient, institution)
Model	22	Detailed description of model, including inputs, outputs, and all intermediate layers and connections
	23	Software libraries, frameworks, and packages
	24	Initialization of model parameters (e.g., randomization, transfer learning)
Training	25	Details of training approach, including data augmentation, hyperparameters, and number of models trained
	26	Method of selecting the final model
	27	Ensembling techniques, if applicable
Evaluation	28	Metrics of model performance
	29	Statistical measures of significance and uncertainty (e.g., confidence intervals)
	30	Robustness or sensitivity analysis
	31	Methods for explainability or interpretability (e.g., saliency maps), and how they were validated
	32	Validation or testing on external data
Results		
Data	33	Flow of participants or cases, using a diagram to indicate inclusion and exclusion
	34	Demographic and clinical characteristics of cases in each partition
Model performance	35	Performance metrics for optimal model(s) on all data partitions
	36	Estimates of diagnostic accuracy and their precision (such as 95% confidence intervals)
	37	Failure analysis of incorrectly classified cases
Discussion		
	38	Study limitations, including potential bias, statistical uncertainty, and generalizability
	39	Implications for practice, including the intended use and/or clinical role
Other information		
	40	Registration number and name of registry
	41	Where the full study protocol can be accessed
	42	Sources of funding and other support; role of funders
